# Efficacy and Safety of First-Line Treatment Strategies for Anaplastic Lymphoma Kinase-Positive Non-Small Cell Lung Cancer: A Bayesian Network Meta-Analysis

**DOI:** 10.3389/fonc.2021.754768

**Published:** 2021-11-08

**Authors:** Ling Peng, Dafeng Lu, Yang Xia, Shaodong Hong, Giovanni Selvaggi, Justin Stebbing, Yilan Sun, Fei Liang

**Affiliations:** ^1^ Department of Respiratory Disease, Zhejiang Provincial People’s Hospital, Affiliated People’s Hospital, Hangzhou Medical College, Hangzhou, China; ^2^ School of Public Health, Nanjing Medical University, Nanjing, China; ^3^ Key Laboratory of Respiratory Disease of Zhejiang Province, Department of Respiratory and Critical Care Medicine, The Second Affiliated Hospital, School of Medicine, Zhejiang University, Hangzhou, China; ^4^ Department of Medical Oncology, Sun Yat-sen University Cancer Center, Guangzhou, China; ^5^ Xcovery Holdings, Palm, Beach Gardens, FL, United States; ^6^ Division of Cancer, Department of Surgery and Cancer, Imperial College London, London, United Kingdom; ^7^ Department of Biostatistics, Zhongshan Hospital, Fudan University, Shanghai, China

**Keywords:** non-small cell lung cancer, ALK, tyrosine kinase inhibitor, network meta-analysis, first-line

## Abstract

**Background:**

Targeted therapies have led to significant improvement in the management and prognosis of anaplastic lymphoma kinase (ALK)-positive non-small cell lung cancer (NSCLC). We performed a network meta-analysis of frontline treatment options of ALK-positive NSCLC to provide clinical guidance.

**Methods:**

PubMed, Embase, ClinicalTrials.gov, and international conference databases were searched to identify relevant trials from inception to June 30, 2021. Phase III randomized controlled trials (RCTs) comparing treatments for patients with ALK-positive advanced NSCLC in the first-line setting were included in a Bayesian network meta-analysis. Eligible studies reported at least one of the following clinical outcomes: progression-free survival (PFS), overall survival (OS), risk of the central nervous system (CNS) progression, adverse events (AEs) of grade (G) 3 or higher (G3 AEs), or serious AEs (SAEs). Hazard ratios (HRs) and CI for primary outcome of PFS and secondary outcome of OS and risk of CNS progression were obtained. A multivariate, consistency model, fixed-effects analysis was used in the network meta-analysis. Data on G3 AEs and SAEs were abstracted and meta-analyzed. Risk of bias (RoB) was assessed using the Cochrane Collaboration’s tool.

**Results:**

Nine RCTs comprising 2,484 patients were included with seven treatments: alectinib, brigatinib, ceritinib, crizotinib, ensartinib, lorlatinib, and chemotherapy. Compared with chemotherapy, ALK-tyrosine kinase inhibitors (TKIs) significantly prolong PFS and reduced risk of CNS progression except for ceritinib. Lorlatinib appears superior at reducing risk of CNS progression. None of the ALK-TKIs have a significantly prolonged OS as compared with chemotherapy. Lorlatinib increases the risk of G3 AEs as compared with alectinib (odds ratio 4.26 [95% CrI 1.22 to 15.53]), while alectinib caused the fewest G3 AEs.

**Conclusions:**

Lorlatinib is associated with the highest PFS benefit and lowest risk of CNS progression benefits for patients with advanced ALK-positive NSCLC, compared with other first-line treatments, but with higher toxicity. The implementation of a newer generation of ALK-TKIs in the first-line treatment of ALK-positive NSCLC into current clinical practice is evolving rapidly.

## Introduction

Anaplastic lymphoma kinase (ALK), a member of the insulin receptor tyrosine kinase family (RTK), is encoded by the ALK gene on chromosome 2p23 (1). The fusion between echinoderm microtubule-associated protein-like 4 (EML4) and ALK has been identified in a minority of non-small cell lung cancer (NSCLC) specimens, and ALK rearrangements are found in approximately 3%–7% of cases, more common among patients with a never/light smoking history, with adenocarcinoma histology, with younger age, and are female and in wild-type tumors for EGFR and KRAS (2). Targeted therapies with small-molecule tyrosine kinase inhibitors (TKIs) to ALK have revolutionized the prognosis and management of ALK-positive NSCLC. Over the past few decades, first-line treatments for ALK-positive advanced NSCLC patients have evolved from the chemotherapy to targeted drugs as TKIs.

Currently, multiple-generation ALK-TKIs have been developed, including crizotinib (first generation); alectinib, brigatinib, ceritinib, and ensartinib (second generation); and lorlatinib (third generation). Randomized controlled trials have been conducted by comparing efficacy and safety of first-line treatments for patients with advanced ALK-positive NSCLC. Relative efficacy and safety among multiple first-line treatments have raised debates. We performed a network meta-analysis to investigate efficacy and safety of first-line treatments in patients with advanced ALK-positive NSCLC to inform the optimal clinical choice.

## Materials and Methods

### Search Strategy

This meta-analysis was performed following the Preferred Reporting Items for Systematic Reviews and Meta-Analyses (PRISMA) extension statement for network meta-analysis (**Table S1**). Bayesian network meta-analysis was used because it offers a more straightforward method for conducting probabilistic statements and predictions on the treatment effects. Institutional review board was exempted due to the nature of the review study.

PubMed, Embase, and ClinicalTrials.gov databases were searched to find relevant articles up to June 30, 2021, in all languages using main search terms “NSCLC” and “ALK” within the restriction limit of “randomized controlled trial”. Abstracts of clinical trials from international conferences were also searched (American Society of Clinical Oncology, European Society for Medical Oncology, and World Conference on Lung Cancer). Finally, the reference lists of the relevant articles were checked for additional studies.

### Study Selection

Phase III randomized controlled trials that met the following criteria were included: 1) patients with histologically or cytologically confirmed advanced (stage III/IV/recurrent) NSCLC with ALK rearrangements; 2) two or more different arms of first-line treatments for patients with ALK-positive NSCLC were compared; and 3) at least one of the following clinical outcome measures: progression-free survival (PFS); overall survival (OS); risk of the central nervous system (CNS) progression, defined by CNS progression, was defined as a new CNS lesion or progression of preexisting CNS lesions, compared with baseline; toxicity regarding adverse events (AEs) of grade 3 or higher defined and graded by the National Cancer Institute’s common terminology criteria for AEs and serious AEs (SAEs).

Exclusion criteria included the following: 1) trials only reporting results from a subgroup analysis; 2) ALK-TKIs were used as neoadjuvant/adjuvant/maintenance treatments, or as sequential treatments with chemotherapy; and 3) treatments that have not been approved by any regulator such as the US Food and Drug Administration. Updated data from long term follow-up were used.

### Data Extraction and Risk of Bias Assessment

Data (e.g., first author, publication year, and patient characteristics), treatments, and reported outcomes were extracted. Survival data were extracted assessed by two independent authors (LP and KX) to avoid potential assessment bias. Risk of bias was assessed using the Cochrane Risk of Bias Tool, including the following domains: random sequence generation, allocation concealment, blinding of participants and personnel, blinding of outcome assessment, incomplete outcome data, selective outcome reporting, and other sources of bias. Items were scored as low, high, or unclear risk of bias. All investigators independently conducted study selection and data extraction. Two investigators (LP and KX) independently assessed risk of bias of individual studies. Any discrepancies were resolved by consensus and arbitration by the authors (LP, KX, YX, FL, and DL).

### Data Synthesis and Statistical Analysis

We synthesized evidence to compare different treatments in terms of efficacy and safety, reported as hazard ratios (HRs) for survival outcomes (PFS, OS, and risk of CNS progression) and odds ratios (ORs) for binary outcomes (G3 AEs and SAEs) along with corresponding 95% credible intervals. The primary outcome was PFS. Secondary outcomes were OS, risk of CNS progression, and G3 AEs and SAEs as reported by the study authors.

Stata (version 14.0) was used to generate network plots to illustrate the geometries, to clarify which treatments were compared directly or indirectly in the included studies (3). Frequentist, fixed-effects, pairwise meta-analysis was performed on head-to-head comparisons. Heterogeneity between studies was assessed using the Q test and *I* ^2^ statistic within a visual forest plot. p-Value of 0.05 was set as statistical significance. Heterogeneity was considered low, moderate, or high for estimated *I* ^2^ values under 25%, between 25% and 50%, and over 50%, respectively.

Network meta-analyses were performed in a Bayesian framework using a Markov chain Monte Carlo simulation technique in R (version 4.0.2). The fixed-effects consistency model was used. For PFS, OS, and risk of CNS progression, 30,000 sample iterations were generated with 20,000 burn-ins and a thinning interval of 1. Convergence of iterations was evaluated by visual inspection of the four chains to establish homogenous parameter estimates and in accordance with the Brooks–Gelman–Rubin diagnostic (**Figure S1**). Once convergence was established, the posterior distributions for the model parameters were obtained as the output of the network meta-analysis estimate (HR/OR and the corresponding 95% credible interval). In the presence of minimally informative priors, credible intervals can be interpreted like conventional CIs. Network meta-analysis estimated the overall rankings of treatments by calculating the surface under the cumulative ranking curve for each, which equals 1 when a treatment is the best and 0 when a treatment is the worst. Transitivity was evaluated using descriptive statistics for study and population baselines, such as sample size, age, and gender. Inconsistency was evaluated by comparing the fit of consistency in models.

## Results

### Study Selection and Characteristics

**Figure 1:**
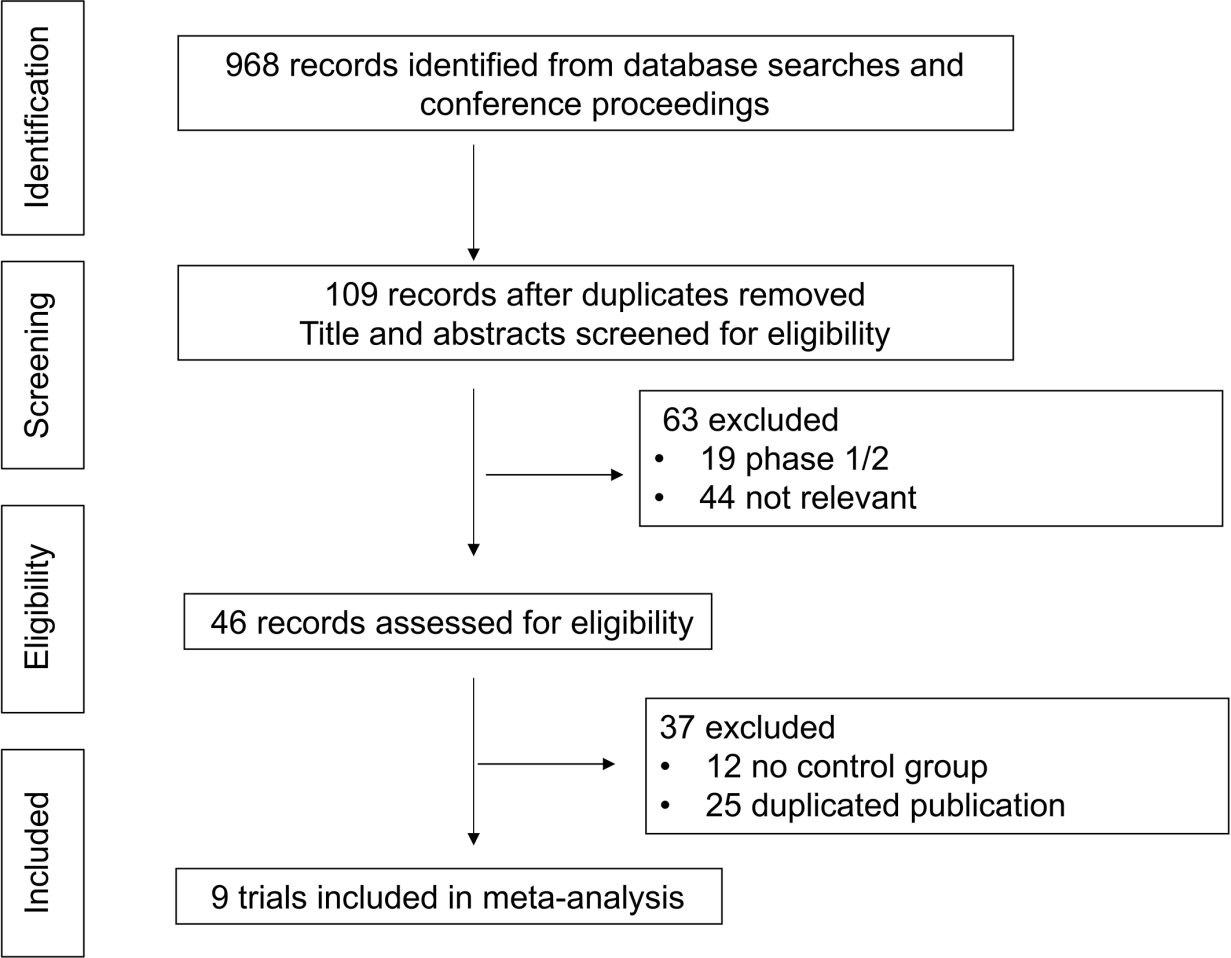
Selection process for the trials included in the meta-analysis. PRISMA diagram. NSCLC, non-small cell lung cancer; RCT, randomized controlled trial; PRISMA, Preferred Reporting Items for Systematic Reviews and Meta-Analyses.

**Figure 2:**
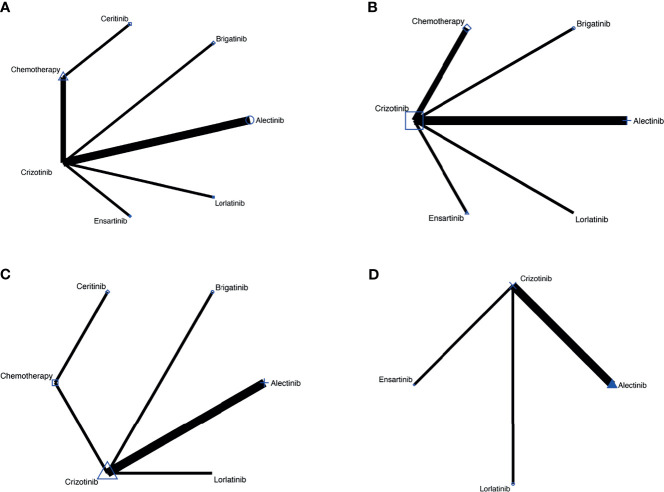
**(A)** Comparisons on PFS and OS in patients with advanced ALK-positive NSCLC. **(B)** Comparisons on risk of CNS progression in patients with advanced ALK-positive NSCLC **(C)** Comparisons on adverse events of grade 3 or higher in patients with advanced ALKpositive NSCLC. **(D)** Comparisons on serious adverse events with advanced ALK-positive NSCLC.

**Figure 3:**
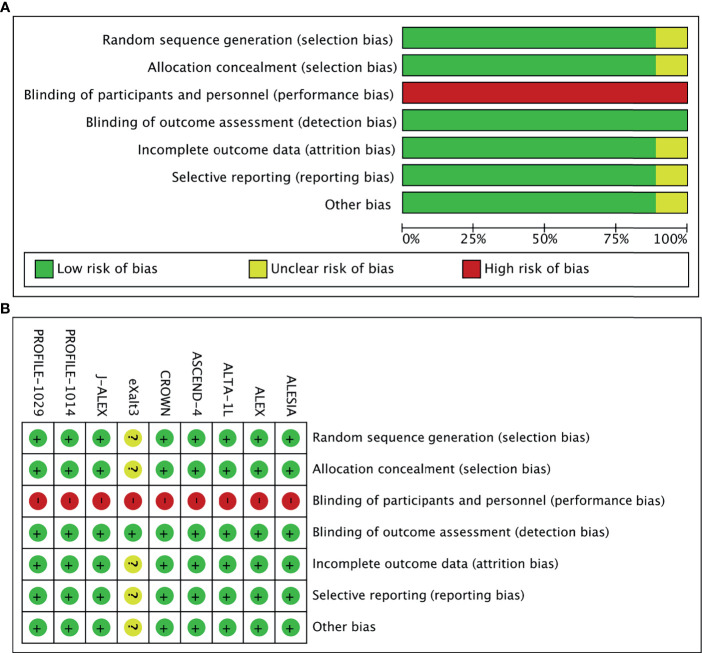
**(A)** Risk-of-bias graph: Each risk-of-bias item presented as percentages across all included studies. **(B)** Risk-of-bias summary: Each risk-of-bias item for each included study.

In total, 968 records were identified from the initial title and abstract screening, and 46 reports were retrieved and reviewed in full text (**Figure 1**). Nine randomized controlled trials were deemed eligible for inclusion with a total of 2,484 patients enrolled to receive seven different treatments including ALK-TKIs (crizotinib, alectinib, brigatinib, ceritinib, ensartinib, or lorlatinib) or chemotherapy. The networks are presented in **Figure 2**. The main characteristics of included studies are reported in **Table 1**. The assumption of transitivity is accepted because no variability was identified in the study and population baselines. The majority of trials include random sequence generation. Overall, the studies are deemed to be at low risk of biases. **Figure 3** summarizes the detailed risk of bias assessments.

**Table 1:** Main characteristics and results of the eligible studies.

Study	Publication/meeting	Phase	Region	Treatment	Control	Sample size (treatment/control)	Median age(years)	Female(%)	Reported outcomes
ALEX (4)	2017 *NEJM*	3	Global	Alectinib	Crizotinib	152/151	58/54	55/58	PFS, OS, risk of CNS progression, G3 AE, SAE
J-ALEX (5)	2017 *Lancet*	3	Japan	Alectinib	Crizotinib	103/104	61.0/59.5	60/61	PFS, OS, risk of CNS progression, G3 AE, SAE
ALESIA (6)	2019 *Lancet Respir Med*	3	Asia	Alectinib	Crizotinib	125/62	51/49	49/45	PFS, OS, risk of CNS progression, G3 AE, SAE
ALTA-1L (7)	2018 *NEJM*	3	International	Brigatinib	Crizotinib	137/138	58/60	50/59	PFS, OS, risk of CNS progression, G3 AE
ASCEND-4 (8)	2017 *Lancet*	3	Global	Ceritinib	Chemotherapy	189/187	55/54	54/61	PFS, OS, G3 AE
PROFILE 1014 (9)	2014 *NEJM*	3	Global	Crizotinib	Chemotherapy	172/171	52/54	63/60	PFS, OS, risk of CNS progression, G3 AE
PROFILE 1029 (10)	2018 *JTO*	3	Asia	Crizotinib	Chemotherapy	104/103	48/50	51.9/58.3	PFS, OS, risk of CNS progression
eXalt3 (11)	2020 *WCLC*	3	Global	Ensartinib	Crizotinib	143/147	54/53	50/48	PFS, OS, risk of CNS progression, SAE
CROWN (12)	2020 *NEJM*	3	Global	Lorlatinib	Crizotinib	149/147	61/56	56/62	PFS, OS, risk of CNS progression, G3 AE, SAE

Summary table of studies included in the meta-analysis.

OS, overall survival; PFS, progression-free survival; CNS, central nervous system; G3 AE, adverse events of grade 3 or higher; SAE, serious adverse event.

### Network Meta-Analysis in Advanced ALK-Positive NSCLC

The network meta-analysis included all treatments for PFS and OS (**Figure 2A**), six treatments for risk of CNS progression (**Figure 2B**), six treatments for G3 AEs (**Figure 2C**), and four treatments for SAEs (**Figure 2D**).

**Figure 4:**
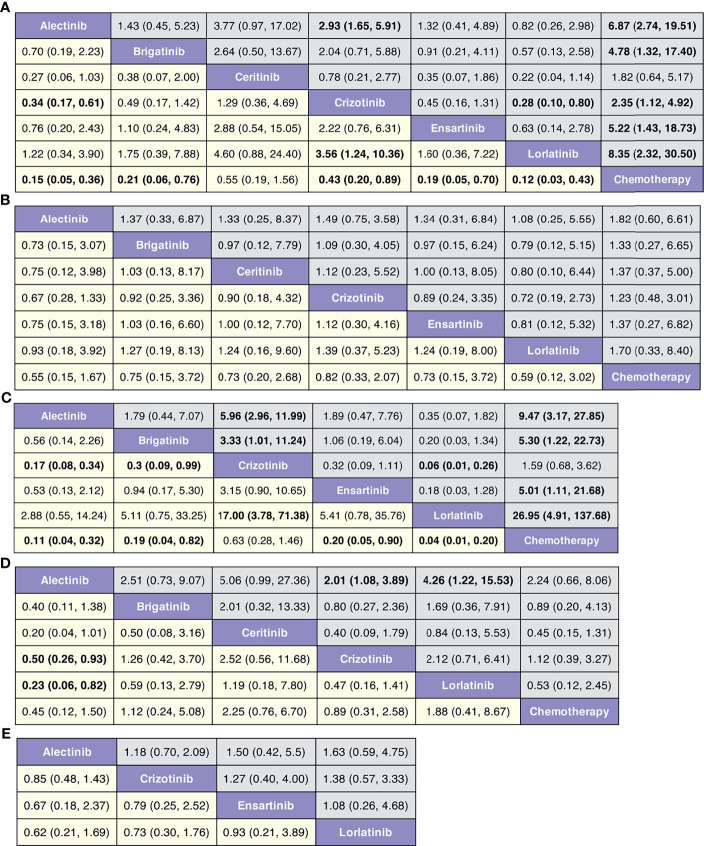
**(A)** Pooled hazard ratios (95% credible intervals) for PFS. **(B)** Pooled hazard ratios (95% credible intervals) for OS. **(C)** Pooled hazard ratios (95% credible intervals) for risk of CNS progression. **(D)** Pooled odds ratios (95% credible intervals) for adverse events of grade 3 or higher. **(E)** Pooled odds ratios (95% credible intervals) for serious adverse events.

In terms of PFS (**Figure 4A** and **Figure S2**), lorlatinib yields the highest benefit versus chemotherapy (HR 0.12, 95% credible interval 0.03 to 0.43), but also significant benefits versus crizotinib (0.28, 0.10 to 0.80). Benefit is also observed with alectinib (0.15, 0.05 to 0.36), ensartinib (0.19, 0.05 to 0.70), brigatinib (0.21, 0.06 to 0.76), and crizotinib (0.43, 0.20 to 0.89), all versus chemotherapy. Alectinib significantly prolongs PFS as compared with crizotinib (0.34, 0.17 to 0.61). In terms of OS (**Figure 4B** and **Figure S3**), none of the ALK-TKIs showed significant differences when compared with chemotherapy or with other ALK-TKI.

In terms of risk of CNS progression (**Figure 4C** and **Figure S4**), lorlatinib is consistent (HR 0.04, 0.01 to 0.20) in providing the highest benefit compared with chemotherapy; significant difference is also observed when compared with crizotinib (0.06, 0.01 to 0.26). Similar efficacy is observed with alectinib versus chemotherapy (0.11, 0.04 to 0.32) and crizotinib (0.30, 0.09 to 0.99).

We observe similar toxicity related to ALK-TKIs among the comparable treatments versus chemotherapy (**Figure 4D** and **Figure S5**). Lorlatinib has higher AEs of grade 3 or higher, than has alectinib (4.26, 1.22 to 15.53); similar higher incidences are observed with crizotinib versus alectinib (2.01, 1.08 to 3.89). No differences are observed regarding the probability of severe AEs among four treatments (alectinib, crizotinib, ensartinib, and chemotherapy, **Figure 4E** and **Figure S6**).

### Rank Probabilities

**Figure 5:**
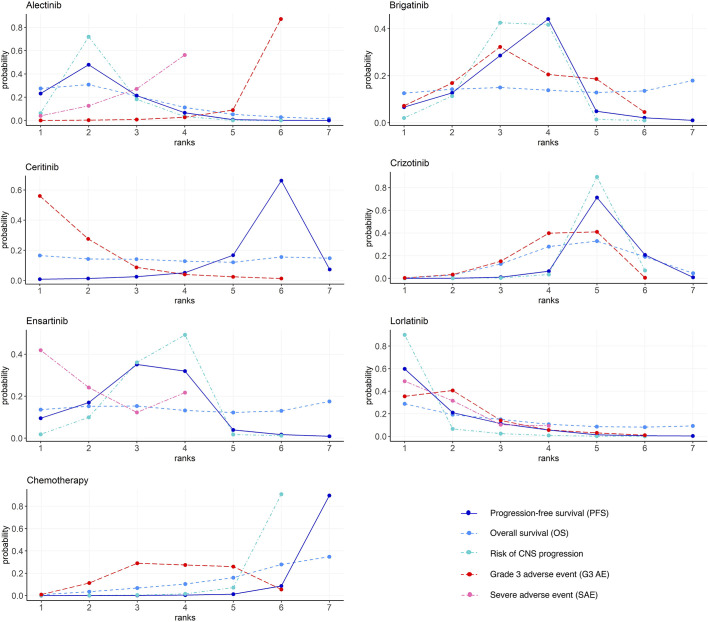
Bayesian ranking profiles of comparable treatments on efficacy for patients with advanced ALK-positive NSCLC. Profiles indicate the probability of each comparable treatment being ranked from first to last on PFS, OS, risk of CNS progression, grade ≥3 adverse events, and serious adverse events. Ranking curves are described according to the Bayesian ranking results presented in **Table S2**. ALK, anaplastic lymphoma kinase; NSCLC, non-small cell lung cancer; PFS, progression-free survival; OS, overall survival; CNS, central nervous system.

**Figure 5** and **Table S2** show the Bayesian ranking profiles of comparable treatments. The Bayesian ranking results are almost in line with the pooled analyses using hazard and ORs. For patients with advanced ALK-positive NSCLC, lorlatinib is most likely to be ranked first for PFS (cumulative probability 60%) and risk of CNS progression (90%).

Ceritinib is most likely to cause G3 AE (56%), followed by lorlatinib (35%), as shown in **Figure 5**. Alectinib has the highest probability (87%) of ranking the last in causing AEs of grade 3 or higher.

### Heterogeneity and Inconsistency Assessment

Forest plots of pairwise comparisons with heterogeneity estimates were generated in **Figure S7**. The results suggest minimal (*I* ^2^ = 0%) heterogeneity in half of all comparisons regarding different outcomes. However, moderate-to-high heterogeneity was detected in the comparisons of crizotinib versus alectinib for PFS (59.2%), OS (74.1%), and AEs of grade 3 or higher (38.1%).

The fit of the consistency model is similar with that of inconsistency model (**Tables S3**). Publication bias was assessed (**Figure S8**).

## Discussion

To our knowledge, the current study represents the most extensive network meta-analysis comparing different treatment options for ALK-positive NSCLC performed to date. In this network meta-analysis, we summarize the comparative efficacy and safety of multiple first-line treatments including all available ALK-TKIs and chemotherapy for patients with advanced ALK-positive NSCLC. The results suggest that lorlatinib ranks the first in providing the PFS benefits and reducing the risk of CNS progression for advanced ALK-positive NSCLC patients. None of the ALK-TKIs perform better than chemotherapy regarding OS based on pairwise comparison. However, in terms of toxicity, ceritinib has the highest rate of G3 AEs followed by lorlatinib.

Since OS is particularly relevant to assess efficacy of treatments (13), this network meta-analysis was conducted to include these results. All OS data included were not mature considering the long median survival for ALK-positive patients. Whether third-generation ALK inhibitors will achieve OS significance when data are mature remains unknown. Most likely, PFS benefits may not translate to OS benefits due to crossover to other ALK-TKIs and or chemotherapy. This is why none of the included trials incorporate OS as primary endpoints. Patients in clinical practice do receive multiple lines of treatment at disease progression, and these contribute to the longer survival of these patients when compared with patients with other types of NSCLC.

Patients with ALK-positive NSCLC have a higher risk of developing brain metastases than patients with other subtypes of NSCLC (14). Given the potentially significant impact of intracranial disease burden on the long-term outcomes of patients with ALK-positive advanced NSCLC, CNS efficacy of ALK-TKIs remains a relevant challenge. Third-generation drugs, such as lorlatinib, were observed to have greater effects on CNS outcomes in terms of reducing the risk of CNS progression.

Differences in toxicity spectrums among ALK-TKIs were observed. The more frequent and severe toxicities of the lorlatinib are hyperlipidemia, edema, and peripheral neuropathy (12). Special AEs to note include CNS effects such as changes in mental status, mood, speech, and sleep. Cognitive effects and mood effects were the most frequently reported treatment-related CNS AEs in patients with or without baseline CNS metastases. Compared with the previously reported network meta-analyses of advanced ALK-positive NSCLC (15, 16), our network meta-analysis has several strengths. Firstly, our study consists exclusively of patients with advanced ALK-positive NSCLC for the first-line treatment, which ensured the homogeneity of study population. Secondly, our study systematically analyzed all major efficacy and toxicity outcomes with the most updated data. Thirdly, although there were only nine trials included, a funnel plot was used to assess the publication bias and small study effects. Moreover, transitivity, heterogeneity, and inconsistency were thoroughly investigated. There are three trials using chemotherapy as comparator arm (8–10), and the drugs used were pemetrexed with platinum; therefore, chemotherapy was grouped as “a single therapy” in our study. However, we did not separate two doses of alectinib, which could be a potential source of heterogeneity and inconsistency, and also possible weak transitivity.

On the other side, our study has several limitations. Firstly, methodologic heterogeneity across studies was anticipated in this network meta-analysis; thus, both pairwise meta-analysis and network meta-analysis were performed to obtain the highest generalizability in the pooled estimates. Secondly, OS data might cause heterogeneity when taken as an endpoint to evaluate each treatment’s effect. Although we initially searched for the most updated OS HRs, data on OS had only 37% maturity in the ALEX trial (17) and 40.8% maturity in the J-ALEX trial (18); thus, it is still tempting for clinicians to consider an improvement in OS benefit for first-line ALK-TKIs compared with conventional chemotherapy. Therefore, we reported PFS as the primary outcome measure. A third limitation was that patients were not stratified according to factors such as ALK variants, drug dose, smoking status, or gender, which might modify treatment benefits. Some existing evidence implies that different variants of ALK rearrangements vary in their clinical and pathological correlations, which suggests that the benefit of ALK-TKIs might differ with variants (19). EML4-ALK fusion variant 3 and TP53 mutation were identified as poor-prognosis biomarkers in ALK+ NSCLC (20). Clinical evidence has also demonstrated different efficacies toward ALK variants. Crizotinib was observed to have better efficacy in patients with ALK variant 1 versus non-variant 1 (21). Ethnic differences in pharmacokinetics of ALK-TKIs were also noted. In the J-ALEX trial (4), Japanese patients received a lower dose (300 mg BID instead of 600 mg BID for western countries) due to the four times lower AUC_0–10_ in US patients than in Japanese patients with ALK-positive NSCLC (22).

There are no available data yet reporting the results of combining ALK-TKI with chemotherapy or anti-angiogenic drugs. Studying combination treatments and potentially different management for subgroups should also be explored for ALK-positive NSCLC patients. Other ALK inhibitors are in development, including repotrectinib (TPX-005), that may represent an effective therapeutic option for patients with ALK-rearranged NSCLC who have progressed on earlier-generation TKIs (23). Furthermore, the role of immune checkpoint inhibitors in ALK-positive NSCLC resistant to ALK-TKIs and chemotherapy is still under investigation (24). Finally, questions regarding the efficacy of treatments in sequential use were not investigated and, therefore, remain a subject for further studies.

## Conclusions

In this network meta-analysis, lorlatinib appears to be superior first-line treatment choices for patients with advanced ALK-positive NSCLC in terms of PFS and risk of CNS progression. We also found that alectinib is associated with the least toxicity and ranked second in PFS and risk of CNS progression.

By synthesizing all randomized controlled evidence, this review provides clinicians a reference source to evaluate strengths and weaknesses for practice choice among multiple promising options.

## Data Availability Statement

The original contributions presented in the study are included in the article/**Supplementary Material**. Further inquiries can be directed to the corresponding authors.

## Author Contributions

Concept and design: YX, FL, and LP. Acquisition, analysis, or interpretation of data: all authors. Drafting of the manuscript: all authors. Critical revision of the manuscript: all authors. Administrative, technical, or material support: LP, DL, FL, and YX. Supervision: LP and FL. All authors contributed to the article and approved the submitted version.

## Conflict of Interest

JS, the Editor-in-Chief of *Oncogene*, has sat on SABs for Vaccitech, Heat Biologics, Eli Lilly, Alveo Technologies, Pear Bio, Agenus, Équilibre BioPharmaceuticals, Graviton Bioscience Corporation, Celltrion, Volvox, Certis Oncology Solutions, Greenmantle, Zedsen, BryoLogyx, and BenevolentAI. He has consulted with Lansdowne Partners and Vitruvian. He sits on the Board of Directors for Xerion and BB Biotech Healthcare Trust PLC. GS is employed by, and holds stock in, Xcovery Holdings, Inc.

The remaining authors declare that the research was conducted in the absence of any commercial or financial relationships that could be construed as a potential conflict of interest.

## Publisher’s Note

All claims expressed in this article are solely those of the authors and do not necessarily represent those of their affiliated organizations, or those of the publisher, the editors and the reviewers. Any product that may be evaluated in this article, or claim that may be made by its manufacturer, is not guaranteed or endorsed by the publisher.
