# Supplementary figures and images for: Efficacy and Safety of First-Line Treatment Strategies for Anaplastic Lymphoma Kinase-Positive Non-Small Cell Lung Cancer: A Bayesian Network Meta-Analysis
Source: Front Oncol. 2021 Nov 8;11:754768. doi: 10.3389/fonc.2021.754768 (PMC8606689; doi:10.3389/fonc.2021.754768)

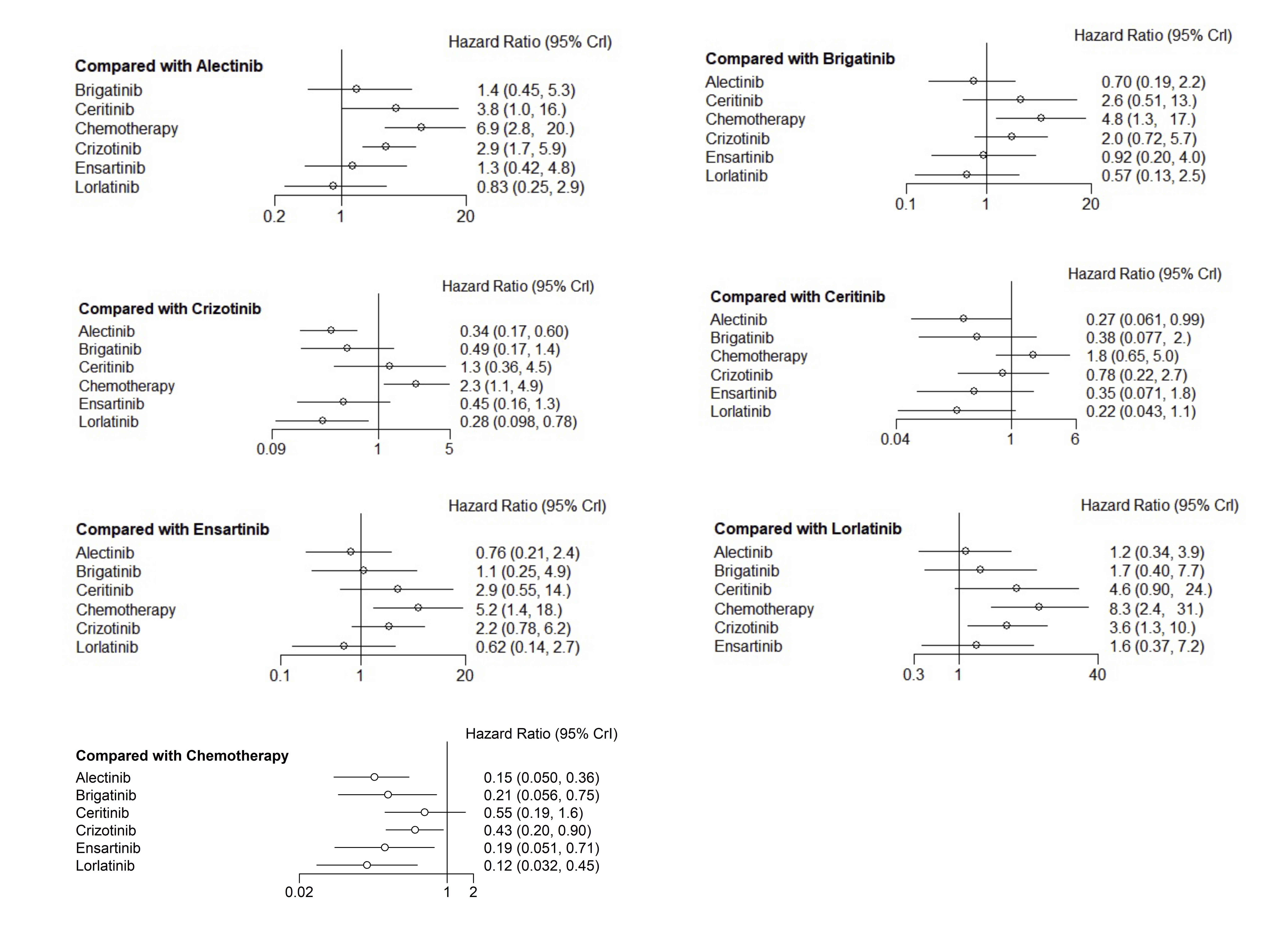

Supplement: Supplementary file 3 [file Image_2.jpeg]

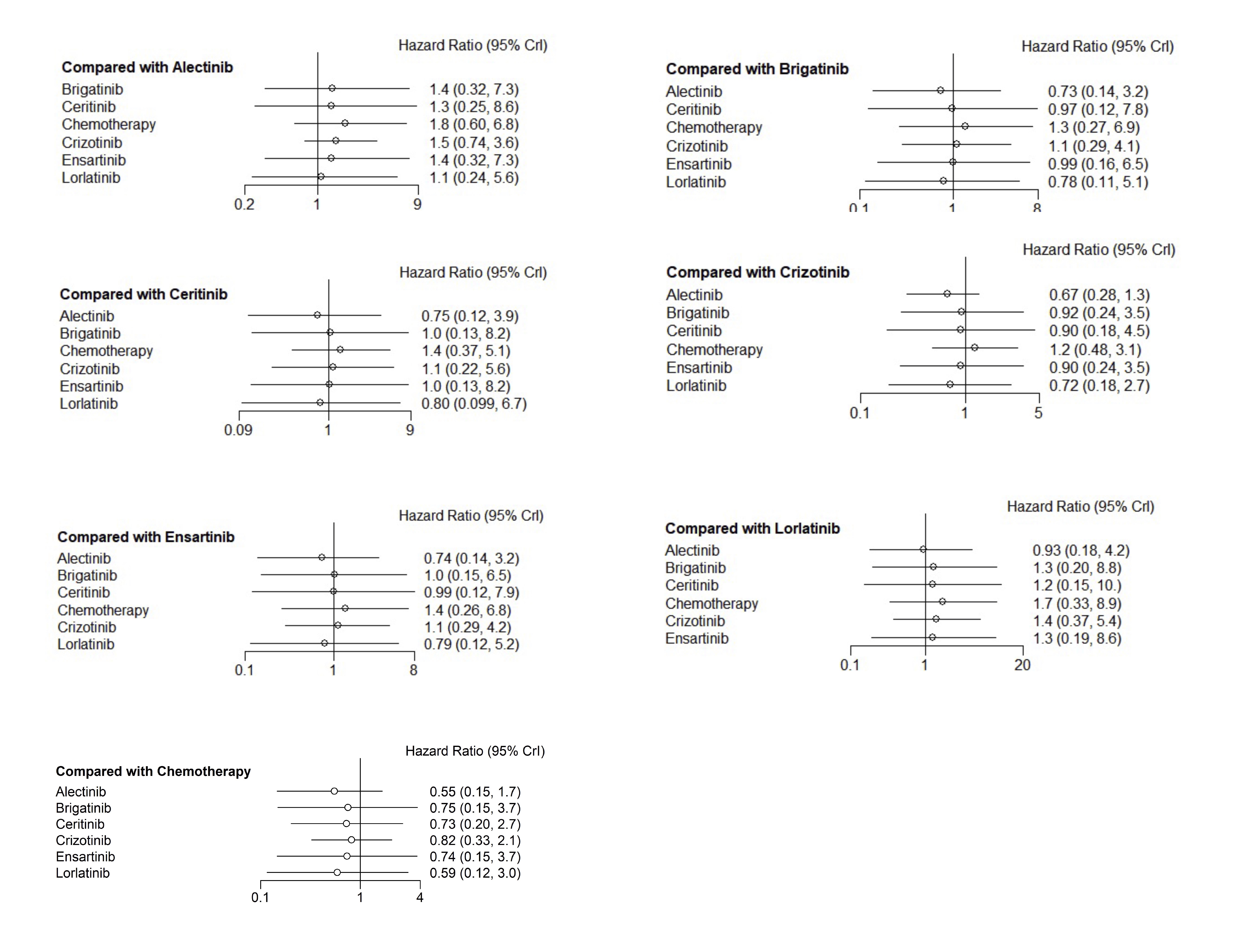

Supplement: Supplementary file 4 [file Image_3.jpeg]

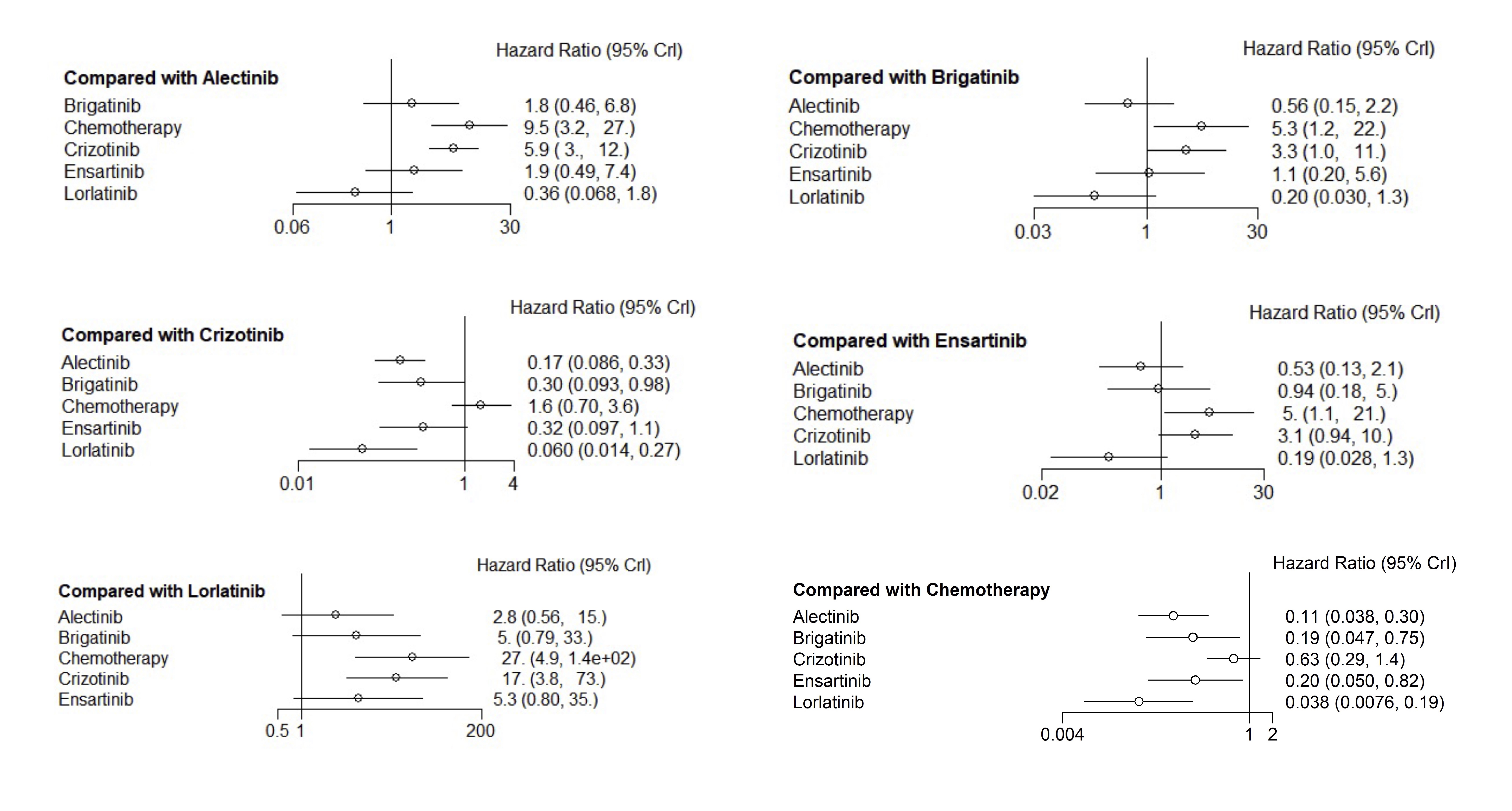

Supplement: Supplementary file 5 [file Image_4.jpeg]

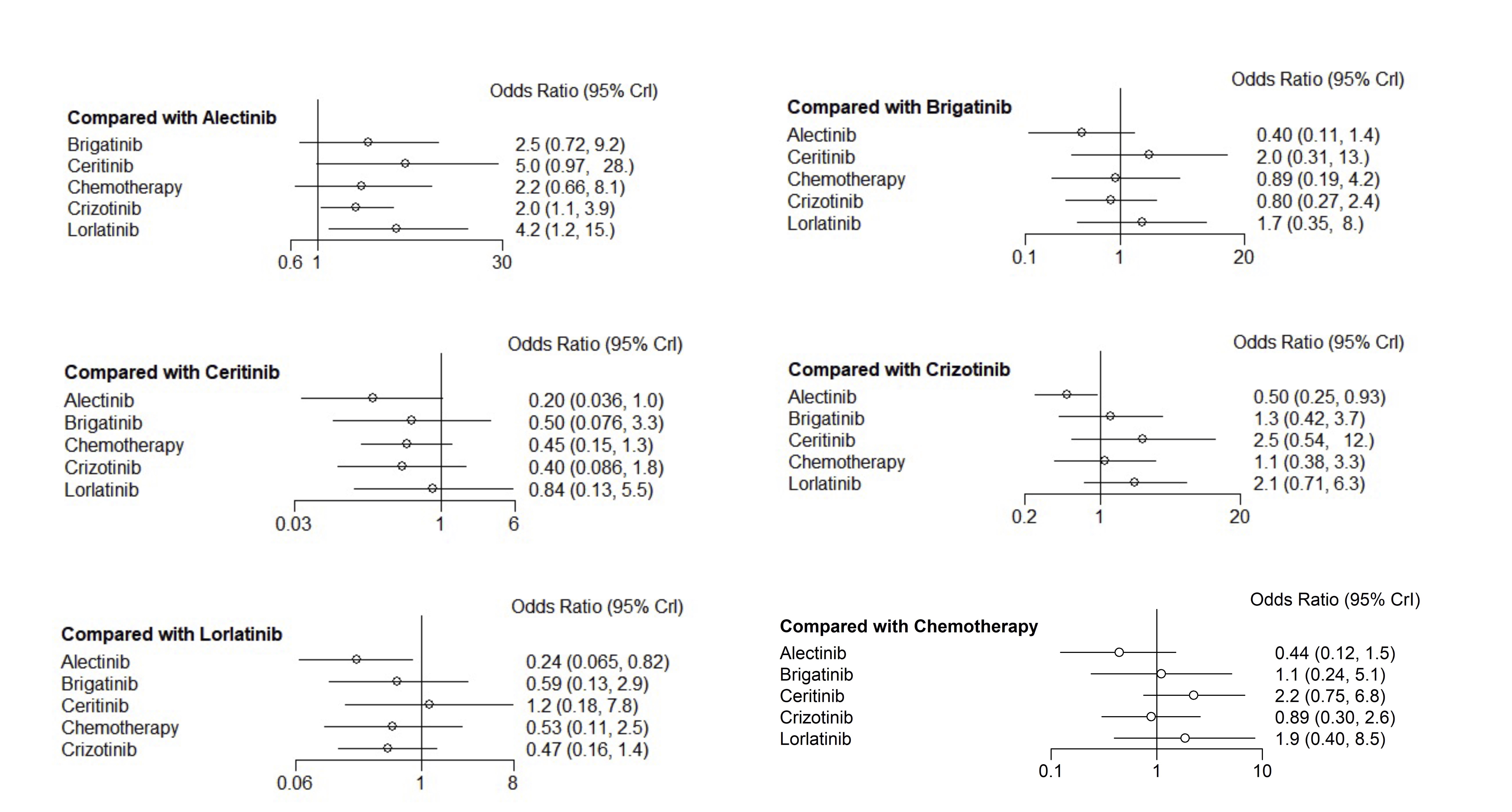

Supplement: Supplementary file 6 [file Image_5.jpeg]

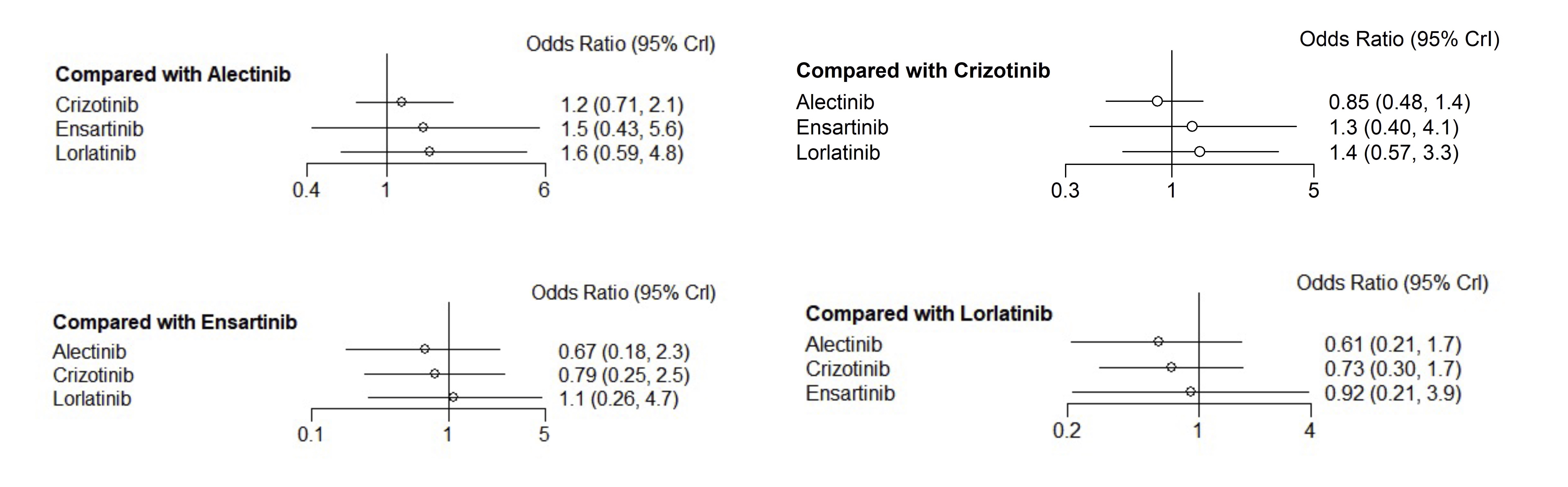

Supplement: Supplementary file 7 [file Image_6.jpeg]

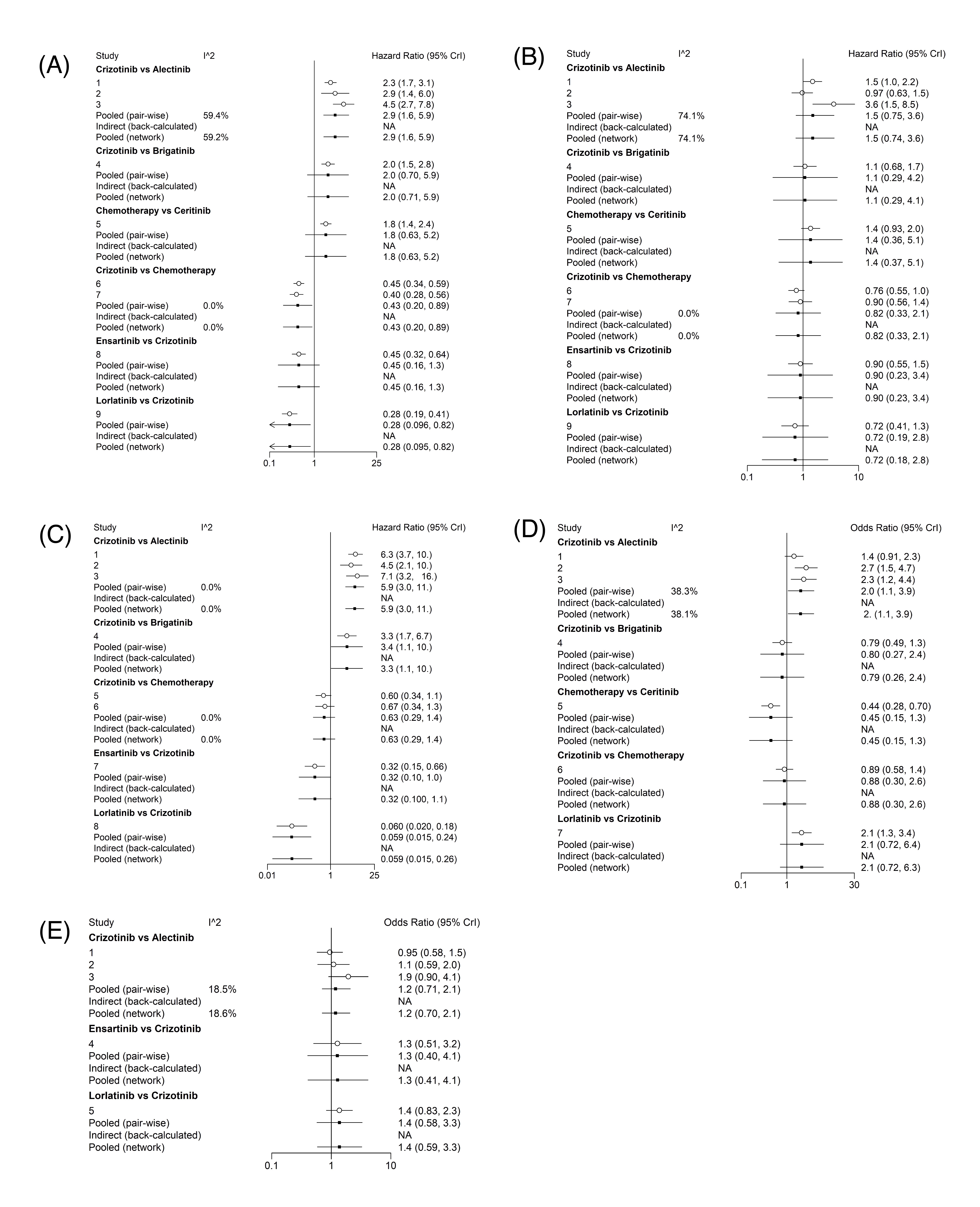

Supplement: Supplementary file 8 [file Image_7.jpeg]

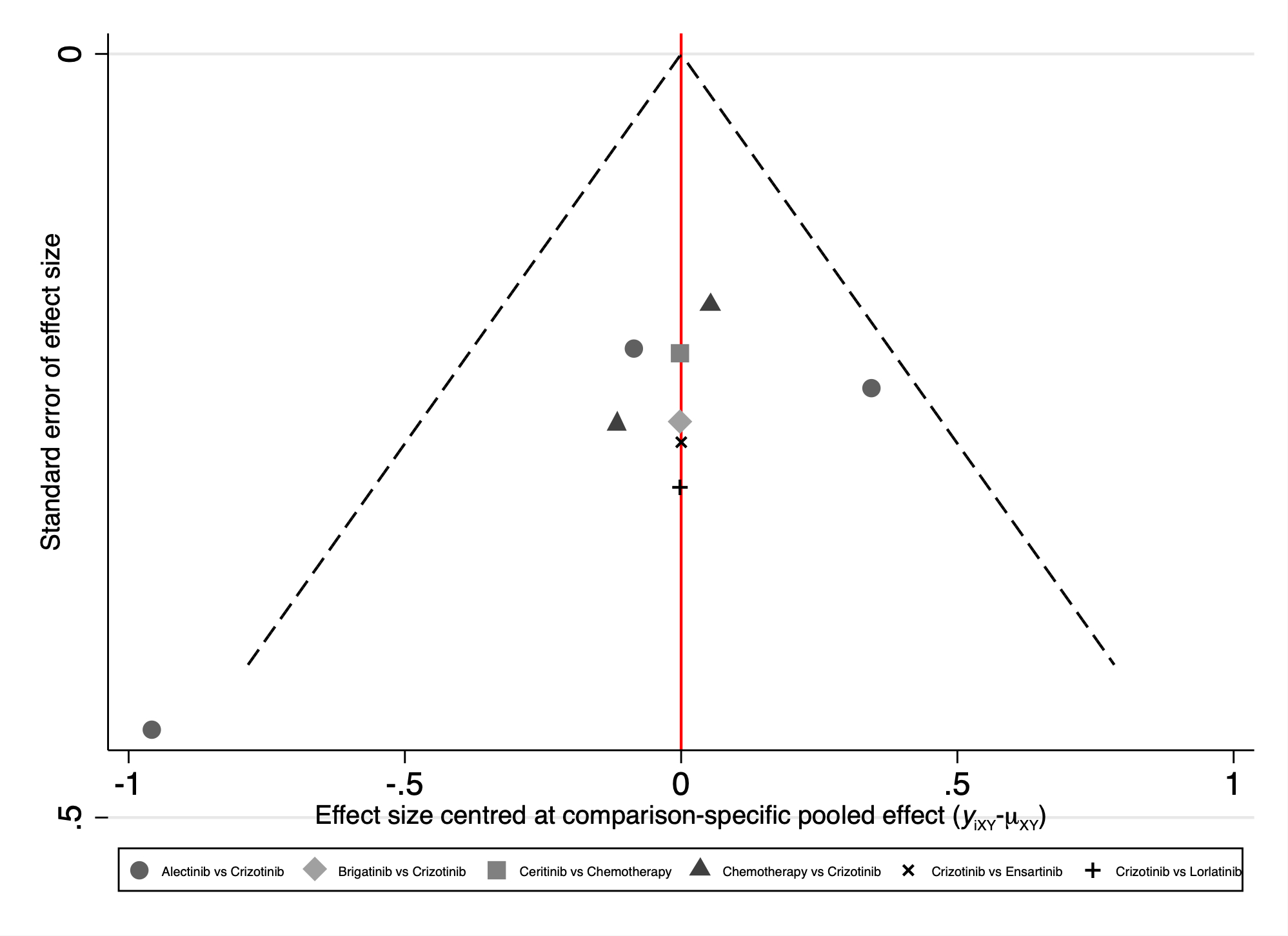

Supplement: Supplementary file 9 [file Image_8.jpeg]
